# Potential of TCR sequencing in graft-versus-host disease

**DOI:** 10.1038/s41409-022-01885-2

**Published:** 2022-12-07

**Authors:** Manisha Goel, Anne Eugster, Johannes Schetelig, Ezio Bonifacio, Martin Bornhäuser, Cornelia S. Link-Rachner

**Affiliations:** 1 Center for Regenerative Therapies Dresden, TU Dresden, Dresden, Germany; 2 Medizinische Klinik und Poliklinik I, Universitätsklinikum Carl Gustav Carus, TU Dresden, Dresden, Germany

**Keywords:** Graft-versus-host disease, Allotransplantation, Haematological cancer, Clonal selection

## Abstract

Graft-versus-host disease (GvHD) remains one of the major complications following allogeneic haematopoietic stem cell transplantation (allo-HSCT). GvHD can occur in almost every tissue, with the skin, liver, and intestines being the mainly affected organs. T cells are implicated in initiating GvHD. T cells identify a broad range of antigens and mediate the immune response through receptors on their surfaces (T cell receptors, TCRs). The composition of TCRs within a T cell population defines the TCR repertoire of an individual, and this repertoire represents exposure to self and non-self proteins. Monitoring the changes in the TCR repertoire using TCR sequencing can provide an indication of the dynamics of a T cell population. Monitoring the frequency and specificities of specific TCR clonotypes longitudinally in different conditions and specimens (peripheral blood, GvHD-affected tissue samples) can provide insights into factors modulating immune reactions following allogeneic transplantation and will help to understand the underlying mechanisms mediating GvHD. This review provides insights into current studies of the TCR repertoire in GvHD and potential future clinical implications of TCR sequencing.

## Introduction

Allogeneic haematopoietic stem cell transplantation (allo-HSCT) is a well-established treatment procedure for haematologic diseases such as high-risk acute leukaemia. Allo-HSCT involves the transfer of haematopoietic stem cells (HSCs) obtained from related or unrelated donors to patients. Donor HSC engraftment and the transferred donor immune system contribute to the eradication of remaining malignant cells. This desired immune reaction is the graft-versus-leukaemia effect [1]. However, a major complication following allo-HSCT is graft-versus-host disease (GvHD). Per definition, acute GvHD (aGvHD) usually occurs within the first months following transplantation. Chronic GvHD (cGvHD) has different clinical features and may occur de novo after days 80–100 or develop as secondary disease in patients with acute GvHD. Both types of GvHD may overlap clinically, leading to relevant mortality, and cGvHD can greatly affect the quality of life of long-term survivors after transplantation. The extent of GvHD can vary from mild signs of inflammation to severe and life-threatening forms in advanced GvHD [2, 3].

**Fig. 1:**
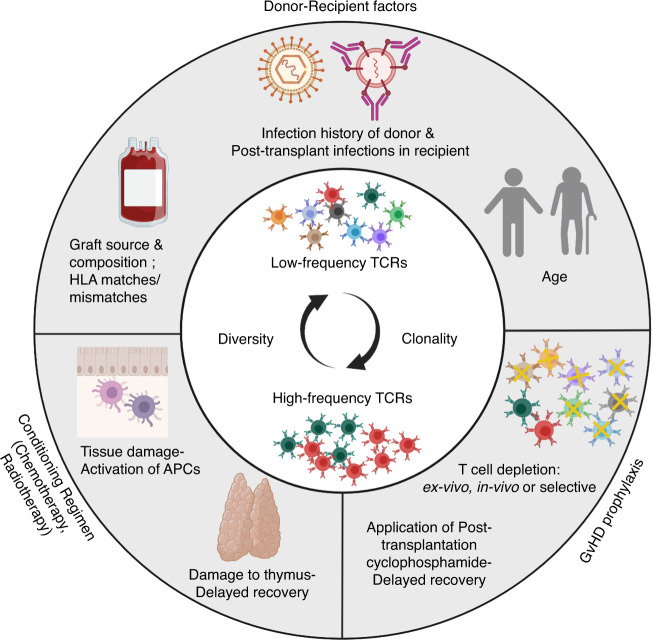
Factors involved in the pathophysiology of Graft-versus-host disease (GvHD) and allogeneic stem cell transplantation (allo-SCT) altering the T cell receptor (TCR) repertoire. The factors altering TCR dynamics post allo-SCT can be divided into donor-recipient factors, the GvHD prophylaxis post-transplant and the conditioning regimen prior to transplant. Donor–recipient factors include transplant regimen, source of graft and HLA matching status between donor and recipient. Other factors are the history of infections of the donor as well as the post-transplant infections of the recipient and the age of both, donor and recipient. They all influence the diversity of the recipient TCR repertoire [8–11]. Infections post transplant influence the TCR diversity due to an increase in antigen-specific TCRs [30, 31]. GvHD prophylaxis requires immunosupressive therapies that influence the TCR repertoire, by depleting T cells [7, 8]. Conditioning regiment involving chemotherapy and radiotherapy leads to the damage of the thymus. This delays thymic recovery influences the dynamics of immune reconstitution and thus the time-dependent recovery of the TCR repertoire. The activation of APCs in recipient tissues is one of the important factors responsible for GvHD [8, 10].

The general concept is that GvHD is initiated by the activation of donor alloreactive T cells, which recognise host-specific histocompatibility antigens presented on host antigen-presenting cells. The activation of donor alloreactive T cells leads to a clonal expansion of selected T cells and to the attraction of other proinflammatory effector cells [4–6]. There is a need to better understand the targets and cellular basis of T cell alloreactivity and its role in the clinical outcome following allo-HSCT [1, 4]. Technologies such as next-generation sequencing (NGS) are promising tools to obtain genetic information and further insights into the T cell pathophysiology of GvHD. Application of NGS to profile dynamics in adaptive immunity includes the adaptive immune receptor repertoire (AIRR) sequencing studies of both, B and T cell receptors. In this review, we focus on T cell receptor (TCR) sequencing studies. The TCR repertoire is likely a footprint of the complex pathophysiology of GvHD, which is shaped by different factors affecting the type of the alloantigen like graft source, HLA matching status, infections, GvHD prophylaxis etc. (Fig. 1) [2, 3, 7–11]. Here, we review the AIRR sequencing studies on T cells in GvHD post HSCT.

## T cells and their T cell receptors in GvHD

T cells have the ability to identify an enormous range of self and foreign epitopes. This ability is due to the presence of heterogeneous antigen receptors on their surface (T cell receptors, TCRs). TCRs are heterodimers that consist of TCR alpha (TRA) and beta (TRB) chains in >95% of T cells and TCR gamma and delta chains in the remaining 5% of T cells [12]. Each chain of the TCR consists of a variable (V) and constant (C) region. The variable region of the TCR is generated by combinatorial somatic rearrangement of variable (V), diversity (D) and joining (J) genes. This somatic rearrangement combined with deletions and additions of non-templated nucleotides at the junctions between the recombining genes leads to the generation of highly diverse TCRs [13]. The variability of the TCR is further enhanced by heterodimer pairing of alpha and beta chains, leading to the number of theoretically possible combinations exceeding 10^19^ [14]. Complementarity-determining region 3 (CDR3), which encompasses the junction of VDJ segments, is mainly responsible for the recognition of and interaction with various antigenic peptides presented by MHC molecules. The other two loops of CDRs—CDR1 and CDR2 encoded by V region are crucial for the interaction between TCR and MHC complex. Upon antigen recognition, assisted by the CD3 zeta chains, T cells and a cascade of signal transducers are activated. This leads to rapid clonal expansion of T cells carrying identical TCRs to generate a population of effector cells [15–17].

The different TCRs within a T cell population define its TCR repertoire. The composition of the TCR repertoire of an individual represents a snapshot of the immunologic state of this individual. The TCR repertoire may quickly change in response to immunologic challenges, and its dynamic nature can be captured only with serial assessments.

In healthy individuals, the TCR repertoire is nonstatic [18]. TCRs can be regarded as molecular identifiers to monitor T cells in different diseases and clinical conditions [14]. There is an unmet need for a method to determine the identity, nature and alloreactivity of T cells present in patients following HSCT [4, 5]. Focusing on the TCR repertoire is therefore considered a promising approach to obtain insights into processes that mediate the GvH reaction.

## TCR repertoire analyses—the past and present

Initially, spectratyping and PCR subcloning techniques were used to assess TCR repertoire diversity. Sanger sequencing also combined with subcloning techniques and spectratyping provides information regarding the CDR3 region of selected TCR clonotypes [14, 19, 20]. While these are useful visual methods to characterise a recipient’s T cell population, they allow only a glimpse of the clonotypes present in the sample analysed. In 2009, NGS was first applied to profile the AIRR [21–24]. AIRR sequencing (AIRRseq) allows high-throughput TCR profiling at high resolution by amplifying and analysing millions of sequences and providing insights into the extent of TCR repertoire diversity [25]. Current AIRRseq techniques use TCR enrichment steps for either genomic DNA or RNA to increase the sensitivity using multiplex PCR or 5′ rapid amplification of cDNA ends (RACE) PCR. However, these bulk approaches to analyse the TCR repertoire are limited in their ability to sequence single TCR chains and thus do not provide information on the pairing of the alpha and beta chains of the TCR [14, 19, 26, 27]. The pairing information of the alpha and beta chains reflects T cells in vivo and can be accurately obtained only through single-cell analysis. Single-cell TCR repertoire analysis can be performed either through TCR sequence reconstruction from single-cell whole RNA sequencing data or by direct, targeted amplification of the TCR from single cells [28]. In human samples, single-cell TCR repertoire analysis for obtaining paired chain information has not been utilised to investigate GvHD yet. Only recently, it was applied for the first time to study T cells dynamics in a mouse model of GvHD [29]. Nevertheless, single-cell sequencing has been applied following allo-SCT to discover CMV-specific clonotypes to get exact pairing information of CMV-specific TCR clonotypes [30, 31].

## TCR diversity in peripheral repertoires of patients affected by GvHD

To characterise peripheral immune reconstitution following allo-HSCT, an evaluation of the TCR repertoire is performed by measuring and comparing features such as the distribution, clonal expansion, and diversity of TCR repertoires in patients. Diversity scores such as Simpson’s diversity index, the Gini coefficient, and Shannon entropy are used to quantify and compare different TCR repertoires and in the general range between maximum diversity and maximum clonality.

The degree of T cell clonal expansion can be estimated with these diversity indices, which are used to characterise the relative distributions of multiple TCR clones (Table 1) [32]. Diversity scores have been used to characterise immune reconstitution of T cells following transplantation under different conditions, e.g., different procedural choices or different graft sources of HSCs, but also in the context of viral infections or GvHD. Studies reporting TCR diversity in GvHD patients are summarised in Table 2.

**Table 1:** Important definitions associated with TCR diversity indices.

Important definitions for TCR diversity analysis
∙ **Gini coefficient:** inequality of a system or frequency distribution of different TCRs with values ranging between 0 (maximum diversity with perfect equality) to 1 (maximum unevenness)
∙ **Shannon entropy & diversity index:** a measure of the randomness within the system, takes into account both species richness and evenness, rises with increase in number of TCRs and evenness of their abundance with 0 representing no diversity. There is no upper limit to the value of Shannon diversity index
∙ **Simpson**’**s diversity index, Ds:** measures the probability that sequences randomly selected from a sample will belong to different T cell clonotype (0 = system with no diversity, 1 = system with high diversity)
∙ **Inverse Simpson**’**s diversity index, 1/(1-Ds):** is inverse of Simpson’s Diversity index (1 = system with no diversity, increasing value = increase in the diversity)

**Table 2:** TCR sequencing studies analysing GvHD tissue biopsy samples and peripheral blood samples.

Author, Journal, Year	Study Design	Mentioned GvHD prophylaxis	Samples & Timepoints (*n* = Number of patients for sequencing)	Measurement of Diversity	Keypoints
Meyer et al., Blood. 2013 [49]	Comparison on basis of response to steroids	CSA/MMF/MTX/Tac	PB—time of aGvHD diagnosis and 25–35 days after; GI biopsy - time of aGvHD diagnosis (*n* = 15)	Gini coefficient	Between the steroid-sensitive and steroid-refractory GvHD patients, there was no difference in TCR diversity in either blood samples or biopsies
					Longitudinal tracking of TCR clones identified in the GI tract in blood at the time of diagnosis and 30 days after indicated clonal expansion in patients with steroid-refractory GvHD when compared with patients with steroid-sensitive GvHD.
					Samples from patients with severe steroid-refractory GvHD had more consistent TRB clonotype populations in different areas of biopsies than those from patients with steroid-sensitive GvHD.
van Heijst et al., Nature Medicine. 2013 [34]	Comparison of TCR diversity in patients with graft source, GvHD status, steroid therapy and infection (CMV/EBV)	T cell depleted graft or CSA/MMF/Tac	PB (CD4 and CD8 T cells)—either 6 months or 1 year post-SCT (*n* = 28, 18 aGvHD patients)	Inverse Simpson’s diversity index	Patients with aGVHD (grade 2 or 3) are associated with higher TCR diversity than patients without aGvHD and with mild grade 1 aGvHD
Yew et al., Bone Marrow Transplantation. 2015 [36]	Comparison of TCR diversity between type of transplant and GvH response	T cell depleted graft or haplo transplant	PB - before SCT and different time points post (GvHD patients - closest to the date of aGvHD diagnosis) (*n* = 21, 11 aGvHD patients)	Inverse Simpson’s diversity index	Significantly stronger enrichment of the ten most abundant TRB clones in GvHD patients than in non-GvHD patients; very modest difference in TRA with GvHD status
Kanakry et al., JCI Insight. 2016 [44]	Retrospective analysis of 8 aGvHD patients by obtaining skin and/or GI tract biopsies	PTCy or CNI/MTX	PB - different timepoints; Skin biopsies - different timepoints; GI biopsies - time of aGvHD diagnosis (*n* = 8)	-	With few exceptions, dominant clones in blood were infrequently observed in GI tract biopsy samples and almost not at all in skin biopsy samples.
Gkazi et al., Front Immunol. 2018 [33]	Assessment of TCR repertoire in cord blood transplant paediatric patients (median age = 2 years and 1 month)	Not mentioned	PB - different timepoints (*n* = 16, 14 GvHD patients)	Gini coefficient	TCR diversity inversely correlated with GvHD scores - GvHD drives clonal expansion
Link-Rachner et al., Haematologica. 2019 [37]	Comparison between different transplant regimens	ATG or PTCy, CSA + MTX, Tac + MMF	PB (CD8 T cells) - day 60 and day 180 post-SCT (*n* = 25, 16 aGvHD patients & 12 cGvHD patients)	Inverse Simpson’s diversity index	aGvHD—memory CD8—tended towards higher diversity; cGvHD—memory CD8—tended towards lower diversity
Koyama et al., Biology of Blood and Marrow Transplantation. 2019 [50]	Evaluate TCR repertoire in GvHD tissues	Tac + MTX	PB - time of aGvHD diagnosis, and before first-line treatment; Biopsies - same as PB (*n* = 8)	-	Clonal expansion of TCR repertoires in different organs in GvHD.
					Limited overlap of the gut and blood repertoire.
					Organ specificity of TCR repertoires post SCT and in GvHD.
Leick et al., Biol Blood Marrow Transplant. 2020 [41]	Look at longitudinal changes in reconstitution of T cells during first 3 months post-SCT	Tac + MTX	PB - different timepoints (day 15, day 30, day 50 and day 100 post-SCT) (*n* = 99, 40 aGVHD patients)		Increased clonal expansion precedes aGVHD development (on comparison with donors)
Shah et al., Biol Blood Marrow Transplant. 2020 [35]	Comparison on basis of response to steroids and mismatches	Not mentioned	PB - time of aGvHD diagnosis (*n* = 146)	Shannon entropy, Simpson’s diversity index	No difference in TCR diversity between steroid responders and nonresponders among aGvHD patients

The table summarises studies using TCR sequencing to analyse TCR repertoires in GvHD patients. The site and timepoint of the collection of samples are mentioned for each study.

*GI* gastrointestinal, *aGvHD* acute graft-vs.-host-disease, *SCT* stem cell transplantation, *TCR* T cell receptor, *TRB* T cell receptor beta chain, *PTCy* post transplantation cyclophosphamide, *ATG* antithymocyte globulin, *Tac* tacrolimus, *MTX* methotrexate, *CSA* cyclosporine, *MMF* mycophenolate mofetil, *CNI* calcineurin inhibitor, *PB* peripheral blood

Both higher and lower TCR repertoire diversities in patients with GvHD as compared to those without GvHD have been described [32–37]. The individual studies cannot be directly compared due to methodological differences. For example, aGvHD and cGvHD are often not addressed separately. Furthermore, the sampling for TCR analyses is often performed at different time points. Several factors as shown in Fig. 1 can affect the TCR repertoire and thus the TCR diversity post allo-SCT and in GvHD. The donor-recipient compatibility, age of the patient, immunosuppressive therapies as part of GvHD prophylaxis, previous damage to thymus by conditioning regimen are few of the several factors contributing to the dynamics of T cell regeneration and repertoire post allo-SCT [7–11, 38–40]. Additionally, conflicting results of TCR diversity could result from differences in the allowed HLA mismatches, which may not have been considered during analysis. It has been shown that patients with permissive HLA-DPB1 mismatches have a greater immunopeptidome overlap between donor and recipient, accompanied by fewer alloreactive TCR clonotypes than their nonpermissive counterparts. This important point needs to be considered in future TCR sequencing studies [11]. In 2013, a study described quantitative assessment of the TRB repertoire following transplantation, comparing patients with aGvHD to patients without GvHD. In TCR diversity analyses of peripheral blood samples from patients with aGvHD (grade 2 or 3) and prior systematic steroid treatment, the TCR diversity was higher in terms of the CD4^+^ and CD8^+^ cell TCR repertoire than in patients without aGvHD and mild grade 1 aGvHD (Table 2). Of note, the diversity was measured 6 months or 1 year after transplantation rather than at the time of a clinical event (i.e. the onset of GvHD, implementation of steroid therapy etc), which allows limited conclusions only. Other studies, however, correlated aGvHD with lower TCR repertoire diversity and with expanded TCR clones (Table 2) [33, 36, 41].

Based on the sequencing of the CDR3 region of the TCRB chain, it was reported that T cell diversity remained low until 100 days post HSCT. The study included several time points, i.e., day 15, day 30, and day 50 post HSCT. When further investigating the relationship between TCR diversity and acute GvHD, the authors found an association between an increased number of expanded T cell clones and acute GvHD, indicating a lower TCR repertoire diversity in these patients [41].

Others did not distinguish between acute and chronic GvHD and reported TCR diversity to be inversely correlated with GvHD scores. The authors suggested that GvHD drives clonal expansions and that this leads to reduced diversity [33]. However, the differentiation between patients with either type of GvHD seems to be important. Differences in the CD8^+^ T cell TRA repertoire diversity between patients with acute and chronic GvHD have been reported. In acute GvHD patients, the TCR repertoire of the memory compartment tended towards higher diversity compared to patients without GvHD. In patients with cGvHD, TCR diversity tended to be lower for the memory repertoire than in patients without GvHD [37]. This tendency implies dynamic changes in the T cell compartments depending on the two types of GvHD, emphasising that aGvHD and cGvHD should be analysed separately. Distinct molecular patterns have been reported in acute and chronic form of cutaneous GvHD [42]. While at early time points post allo-SCT, aGvHD is mediated by matured T cells contained in the graft, cGvHD is dependent on the regeneration of thymic functions and the leakage of autoreactive T cells. This emphasises the importance to separate the diseases when analysing T cell receptor repertoire in GvHD [2, 3, 43].

One key question in GvHD is if the knowledge of TCR diversity during GvHD can be used to predict the response to steroid therapy. A recent post-HSCT analysis attempted to examine the T cell repertoire across aGvHD patients with steroid treatment. In this study, the TCR diversity of steroid responders and non-responders was not different when compared using different diversity indices. The authors also indicated that a small subset of T cell clones was shared among aGvHD patients, especially among those who were refractory to steroid treatment [35]. The study by Kanakry et al. shows that patients with steroid-refractory GvHD have enhanced clonal expansion of gastrointestinal-identified TCR clonotypes in comparison to steroid-sensitive GvHD patients at the time of diagnosis and 30 days post diagnosis in peripheral blood samples [44]. It is yet to be inferred for certainty if diversity during GvHD can predict outcome and therapy response.

## TCR repertoire studies in GvHD-affected tissues

### Early spectratyping and PCR subcloning studies with GvHD tissue analyses

Most studies have used peripheral blood samples as a source to examine TCR repertoires in patients post HSCT and with GvHD. Information about GvHD-mediating clonotypes is, however, more likely obtained by analysing the tissue-specific repertoire. There are few studies analysing the TCR repertoire from GvHD-affected tissues. Early studies using TCR spectratyping and PCR subcloning reported few expanded TCR clones in GvHD tissues forming an oligoclonal repertoire [45–48]. In one of the earlier studies on GvHD, the variable region usage of TRA and TRB chains and their CDR3 sequences were analysed. Differences between the CDR3 diversity of the TRAV and TRBV regions in GvHD tissues and blood samples were observed. The study concluded that TCR clones in blood and GvHD-affected tissues differed from each other in terms of their CDR3 sequence and gene usage. The low sample size of this study restricts the conclusions about the organ specificity of the T-cell populations [46].

In another study, aiming to identify tissue-derived TCR clones as markers of GvHD, TRBV segments from genomic DNA from skin biopsy samples from eight patients were amplified using clonotypic PCR assays. This study reported that the TCR repertoire of seven GvHD skin biopsy samples had immunodominant clonotypes. For one out of the seven patients, the authors looked at the presence of the dominant skin clonotype in the patient´s peripheral blood sample. The dominant skin clonotype was found in the blood sample, and the authors suggested that tissue-derived clonotypes can serve as biomarkers for GvHD if their presence in tissue and blood can be correlated with active disease [45].

### TCR NGS studies with GvHD tissue analyses

NGS studies analysing the TCR repertoire from GvHD-affected tissues are summarised in Table 2. In 2013, the first study sequencing the TRB repertoire from GvHD-affected human tissue samples was published. In this study, the authors have tried to address the differences in response to steroid treatment in GvHD patients using TCR sequencing. TRB was amplified from genomic DNA from gastrointestinal (GI) samples from 15 patients with acute GI GvHD and was sequenced to identify potential GvHD-mediating T cell clonotypes. Each patient had repertoires with very little interindividual overlap. However, different areas of the GI tract of the same individual were found to have a higher similarity in TRB repertoires in patients with severe steroid-refractory GvHD than in patients with steroid-sensitive GvHD. In this study, the authors longitudinally tracked GI-identified TCR clonotypes in peripheral blood samples. It was shown that patients with steroid-refractory GvHD had enhanced clonal expansion of GI-identified TCR clonotypes in comparison to steroid-sensitive GvHD patients at the time of diagnosis and 30 days post diagnosis in peripheral blood samples [49].

In another study, TRB sequencing was used to study the TCR repertoire of skin and/or GI tract biopsy samples from eight GvHD patients. The dominant TCR clones from blood samples were rarely found in GI biopsy samples and almost never in skin biopsy samples, with few exceptions. The authors concluded that the TCR repertoire is compartmentalised and organ specific [44].

In a detailed study to evaluate the TCR repertoire in GvHD tissues by sequencing the TRB repertoires from different tissue biopsies, 12 tissue samples (skin and intestine) and 8 blood samples from 8 transplanted patients with acute GvHD were analysed. The TCR repertoire was more skewed in the biopsy samples than in the samples from peripheral blood. In skin biopsies from one patient, two TCR clonotypes represented 98% of the TCR repertoire of all T cells infiltrating the skin lesions [50]. The findings from this study suggest that the dominant clonotypes differ across tissues within the same patient. Furthermore, clonotypes identified in GvHD tissue were not identified in the concurrent blood sample from the patient. These findings imply that acute GvHD may be mediated by clonotypes that differ between tissue and blood [50]. The correlations between the frequencies and tissue specificities of clonotypes in GvHD-affected tissues and in peripheral blood remain to be clarified. Sequencing depth of the samples from various origins and limited initial starting material (DNA/RNA or cells) can greatly influence the analysis, especially when overlap between GvHD-affected tissue- and blood samples is of interest. Due to the potential local differences, it needs to be clarified if the local TCR repertoire obtained from a biopsy sample is representative of the entire GvHD-affected area. Further, the often low quantity of available starting material limits the amount of clonotypes that can be recovered from the targeted tissue biopsies leading to low repertoire coverage in tissue biopsies, and making ‘missing’ TCR clonotypes to false negatives. It is still early to say if we will be able to overcome the differences in the sequencing depth of tissue and blood repertoires.

So far, bone marrow samples of GvHD patients have not been analysed. To our knowledge, only one study included samples taken from bone marrow aspirates in the context of GvHD including only a sample collected from one donor prior to transplantation and not from the affected patient [44].

Generally, the findings suggest that monitoring the TCR repertoires of peripheral blood is at best an indirect measure of what may be happening at the site of GvHD. Nevertheless, studies remain few, and comprehensive longitudinal studies are needed. Longitudinal immune profiling can help to identify changes in the immune receptor repertoire prior to important clinical events, like the onset of acute or chronic GvHD and beginning resistance to therapies. It can also provide insights into clinically permissive mismatches of minor and major histocompatibility antigens [11]. In addition, clonotypes identified to be expanding over time may be used to identify target antigens and open avenues for further therapeutic development [51].

## Outlook and current limitations

With the emergence of TCR sequencing as a more broadly applicable technique, several major key questions will need to be addressed by future studies to define its clinical potential in GvHD (Table 3). Meaningful clinical application would most likely become possible if TCR diversity can 1) predict the probability of GvHD occurrence or 2) changes in TCR diversity during GvHD can predict outcome or therapy response. Currently, answers to these questions are still open and longitudinal analysis of larger cohorts of patients with and without acute or chronic GvHD stratified for underlying disease, pre-treatment, infections post-transplant, GvHD prophylaxis need to be performed [18, 41, 49].

**Table 3:** Key Questions in the field of TCR sequencing in GvHD.

Key Questions:
Potential clinical applications of TCR sequencing in GvHD
∙ Does TCR diversity predict the probability of GvHD?
∙ Does TCR diversity during GvHD predict outcome and/or therapy response?
∙ Does GvHD directly impact subsequent TCR diversity and does this impact outcome?
∙ Are common clonotypes shared among individuals with GvHD?

A second major challenge is the identification of shared GvHD mediating clonotypes that allow to predict GvHD. Other than the diversity, TCR sequencing data can be used to trace individual clonotypes across time or different tissue samples in an individual. It can also be used to identify overlapping clonotypes across individuals. While it is still early to say if there are any common clonotypes or target epitopes between individuals in GvHD-affected patients, single-cell paired chain TCR sequencing, as a more advanced technique, combined with longitudinal tracking of expanded clonotypes, may help to first identify recurring TCRs and then to investigate their target antigens. Single-cell TCR sequencing gives access to paired TCR information and thus to the whole TCR [28]. Additionally, single-cell gene expression can be combined with the TCR information and thus activation signatures of clonally expanded TCRs induced by a clinical condition can be determined [51]. Identification of such clonotypes and their signatures would shift TCR sequencing from a prognostic tool to a therapeutic application as these clonotypes could be potentially targeted [52].

While there are multiple studies of peripheral blood TCR repertoires in GvHD patients following HSCT, the majority of these studies have limitations that make it difficult to draw final conclusions. First, many studies are based on a limited number of patients and second, there are no existing gold standards for AIRRseq so far. Studies are therefore difficult to compare, due to these differences in input for sequencing, sequencing method, analysis tools used and normalisations performed during analysis. There is a need to develop standards for AIRRseq studies that allow for the comparison of different AIRRseq data, and the AIRR Community has been working towards setting these benchmarks [25, 27, 28].

## Conclusion

TCR sequencing has the potential to improve our understanding of GvHD, but standardised protocols are required before this technique can be implemented into clinical routine. Standardised approaches, together with prospective longitudinal study protocols combining assessments of both tissue and blood, should provide insight into targets and the potential use of TCR repertoires to monitor patients to better understand and treat post-HSCT GvHD and thus have potential to address key scientific and clinical questions in the field of GvHD.

## Data Availability

Data sharing is not applicable to this article as no datasets were generated or analysed during the current study.

## References

[1] van Besien K (2013). Allogeneic transplantation for AML and MDS: GVL versus GVHD and disease recurrence. Hematol Am Soc Hematol Educ Program.

[2] Zeiser R, Blazar BR (2017). Acute graft-versus-host disease—biologic process, prevention, and therapy. N Engl J Med.

[3] Zeiser R, Blazar BR (2017). Pathophysiology of chronic graft-versus-host disease and therapeutic targets. N Engl J Med.

[4] Ichiki Y, Bowlus CL, Shimoda S, Ishibashi H, Vierling JM, Gershwin ME (2006). T cell immunity and graft-versus-host disease (GVHD). Autoimmun Rev.

[5] Amsen D (2017). T cells take directions from supporting cast in graft-versus-host disease. J Clin Investig.

[6] Reddy P, Ferrara JLM. Mouse models of graft-versus-host disease. StemBook. 2008. 20614594

[7] Zhao C, Bartock M, Jia B, Shah N, Claxton DF, Wirk B (2022). Post-transplant cyclophosphamide alters immune signatures and leads to impaired T cell reconstitution in allogeneic hematopoietic stem cell transplant. J Hematol Oncol.

[8] Gomez-Arteaga A, van Besien K (2022). Allogeneic transplant graft source—conditioning—GVHD prophylaxis: don’t mix and match!. Leuk Lymphoma.

[9] Kolb HJ (2017). Hematopoietic stem cell transplantation and cellular therapy. Hla.

[10] Krishna C, Chowell D, Gonen M, Elhanati Y, Chan TA (2020). Genetic and environmental determinants of human TCR repertoire diversity. Immun Ageing.

[11] Meurer T, Crivello P, Metzing M, Kester M, Megger DA, Chen W (2021). Permissive HLA-DPB1 mismatches in HCT depend on immunopeptidome divergence and editing by HLA-DM. Blood..

[12] Morita CT, Mariuzza RA, Brenner MB (2000). Antigen recognition by human gamma delta T cells: pattern recognition by the adaptive immune system. Springe Semin Immunopathol.

[13] Tonegawa S (1983). Somatic generation of antibody diversity. Nature..

[14] Bradley P, Thomas PG (2019). Using T cell receptor repertoires to understand the principles of adaptive immune recognition. Annu Rev Immunol.

[15] Scaviner D, Lefranc MP (2000). The human T cell receptor alpha variable (TRAV) genes. Exp Clin Immunogenet.

[16] Folch G, Lefranc MP (2000). The human T cell receptor beta variable (TRBV) genes. Exp Clin Immunogenet.

[17] Jr CAJ, Travers P, Walport M, Shlomchik MJ, Jr CAJ, Travers P, et al. Immunobiology. 5th ed: Garland Science; 2001.

[18] Chu ND, Bi HS, Emerson RO, Sherwood AM, Birnbaum ME, Robins HS (2019). Longitudinal immunosequencing in healthy people reveals persistent T cell receptors rich in highly public receptors. BMC Immunol.

[19] Six A, Mariotti-Ferrandiz ME, Chaara W, Magadan S, Pham HP, Lefranc MP (2013). The past, present, and future of immune repertoire biology - the rise of next-generation repertoire analysis. Front Immunol.

[20] Nielsen SCA, Boyd SD (2018). Human adaptive immune receptor repertoire analysis—past, present, and future. Immunol Rev.

[21] Boudinot P, Marriotti-Ferrandiz ME, Pasquier LD, Benmansour A, Cazenave PA, Six A (2008). New perspectives for large-scale repertoire analysis of immune receptors. Mol Immunol.

[22] Weinstein JA, Jiang N, White RA, Fisher DS, Quake SR (2009). High-throughput sequencing of the zebrafish antibody repertoire. Science.

[23] Freeman JD, Warren RL, Webb JR, Nelson BH, Holt RA (2009). Profiling the T-cell receptor beta-chain repertoire by massively parallel sequencing. Genome Res.

[24] Glanville J, Zhai W, Berka J, Telman D, Huerta G, Mehta GR (2009). Precise determination of the diversity of a combinatorial antibody library gives insight into the human immunoglobulin repertoire. Proc Natl Acad Sci USA.

[25] Breden F, Luning Prak ET, Peters B, Rubelt F, Schramm CA, Busse CE (2017). Reproducibility and reuse of adaptive immune receptor repertoire data. Front Immunol.

[26] Barennes P, Quiniou V, Shugay M, Egorov ES, Davydov AN, Chudakov DM, et al. Benchmarking of T cell receptor repertoire profiling methods reveals large systematic biases. Nat Biotechnol. 2021;39:236–45. 10.1038/s41587-020-0656-3 32895550

[27] Rosati E, Dowds CM, Liaskou E, Henriksen EKK, Karlsen TH, Franke A (2017). Overview of methodologies for T-cell receptor repertoire analysis. BMC Biotechnol.

[28] Valkiers S, de Vrij N, Gielis S, Verbandt S, Ogunjimi B, Laukens K (2022). Recent advances in T-cell receptor repertoire analysis: bridging the gap with multimodal single-cell RNA sequencing. ImmunoInformatics.

[29] Engel JA, Lee HJ, Williams CG, Kuns R, Olver S, Lansink LI, et al. Single-cell transcriptomics of alloreactive CD4+ T cells over time reveals divergent fates during gut graft-versus-host disease. JCI Insight. 2020;5:13. 10.1172/jci.insight.137990 PMC7406307 32484791

[30] Link CS, Eugster A, Heidenreich F, Rucker-Braun E, Schmiedgen M, Oelschlagel U (2016). Abundant cytomegalovirus (CMV) reactive clonotypes in the CD8(+) T cell receptor alpha repertoire following allogeneic transplantation. Clin Exp Immunol.

[31] Erickson JR, Stevens-Ayers T, Mair F, Edmison B, Boeckh M, Bradley P, et al. Convergent clonal selection of donor- and recipient-derived CMV-specific T cells in hematopoietic stem cell transplant patients. Proc Natl Acad Sci USA. 2022;119:6. 10.1073/pnas.2117031119 PMC8833188 35105810

[32] Greiff V, Miho E, Menzel U, Reddy ST (2015). Bioinformatic and statistical analysis of adaptive immune repertoires. Trends Immunol.

[33] Gkazi AS, Margetts BK, Attenborough T, Mhaldien L, Standing JF, Oakes T (2018). Clinical T cell receptor repertoire deep sequencing and analysis: an application to monitor immune reconstitution following cord blood transplantation. Front Immunol.

[34] van Heijst JW, Ceberio I, Lipuma LB, Samilo DW, Wasilewski GD, Gonzales AM (2013). Quantitative assessment of T cell repertoire recovery after hematopoietic stem cell transplantation. Nat Med.

[35] Shah O, Tamaresis JS, Kenyon LJ, Xu L, Zheng P, Gupta P (2020). Analysis of the whole CDR3 T cell receptor repertoire after hematopoietic stem cell transplantation in 2 clinical cohorts. Biol Blood Marrow Transplant.

[36] Yew PY, Alachkar H, Yamaguchi R, Kiyotani K, Fang H, Yap KL (2015). Quantitative characterization of T-cell repertoire in allogeneic hematopoietic stem cell transplant recipients. Bone Marrow Transplant.

[37] Link-Rachner CS, Eugster A, Rücker-Braun E, Heidenreich F, Oelschlägel U, Dahl A (2019). T-cell receptor-α repertoire of CD8+ T cells following allogeneic stem cell transplantation using next-generation sequencing. Haematologica.

[38] Andrlova H, van den Brink MRM, Markey KA (2020). An unconventional view of T cell reconstitution after allogeneic hematopoietic cell transplantation. Front Oncol.

[39] Dekker L, de Koning C, Lindemans C, Nierkens S. Reconstitution of T cell subsets following allogeneic hematopoietic cell transplantation. Cancers. 2020;12:1974. 10.3390/cancers12071974 PMC7409323 32698396

[40] Aran A, Garrigos L, Curigliano G, Cortes J, Marti M. Evaluation of the TCR repertoire as a predictive and prognostic biomarker in cancer: diversity or clonality? Cancers. 2022;14:1771. 10.3390/cancers14071771 PMC8996954 35406543

[41] Leick M, Gittelman RM, Yusko E, Sanders C, Robins H, DeFilipp Z (2020). T cell clonal dynamics determined by high-resolution TCR-beta sequencing in recipients after allogeneic hematopoietic cell transplantation. Biol Blood Marrow Transpl.

[42] Bruggen MC, Klein I, Greinix H, Bauer W, Kuzmina Z, Rabitsch W (2014). Diverse T-cell responses characterize the different manifestations of cutaneous graft-versus-host disease. Blood..

[43] Martin PJ (2008). Biology of chronic graft-versus-host disease: implications for a future therapeutic approach. Keio J Med.

[44] Kanakry CG, Coffey DG, Towlerton AMH, Vulic A, Storer BE, Chou J, et al. Origin and evolution of the T cell repertoire after posttransplantation cyclophosphamide. JCI Insight. 2016;1:5. 10.1172/jci.insight.86252 PMC4874509 27213183

[45] Beck RC, Wlodarski M, Gondek L, Theil KS, Tuthill RJ, Sobeck R (2005). Efficient identification of T-cell clones associated with graft-versus-host disease in target tissue allows for subsequent detection in peripheral blood. Br J Haematol.

[46] Hirokawa M, Matsutani T, Saitoh H, Ichikawa Y, Kawabata Y, Horiuchi T (2002). Distinct TCRAV and TCRBV repertoire and CDR3 sequence of T lymphocytes clonally expanded in blood and GVHD lesions after human allogeneic bone marrow transplantation. Bone Marrow Transpl.

[47] Margolis DA, Casper JT, Segura AD, Janczak T, McOlash L, Fisher B (2000). Infiltrating T cells during liver graft-versus-host disease show a restricted T-cell repertoire. Biol Blood Marrow Trans.

[48] Kubo K, Yamanaka K, Kiyoi H, Fukutani H, Ito M, Hayakawa R (1996). Different T-cell receptor repertoires between lesions and peripheral blood in acute graft-versus-host disease after allogeneic bone marrow transplantation. Blood..

[49] Meyer EH, Hsu AR, Liliental J, Löhr A, Florek M, Zehnder JL (2013). A distinct evolution of the T-cell repertoire categorizes treatment refractory gastrointestinal acute graft-versus-host disease. Blood..

[50] Koyama D, Murata M, Hanajiri R, Akashi T, Okuno S, Kamoshita S (2019). Quantitative assessment of T cell clonotypes in human acute graft-versus-host disease tissues. Biol Blood Marrow Transplant.

[51] Emerson RO, DeWitt WS, Vignali M, Gravley J, Hu JK, Osborne EJ (2017). Immunosequencing identifies signatures of cytomegalovirus exposure history and HLA-mediated effects on the T cell repertoire. Nat Genet.

[52] Zhao QJ, Jiang Y, Xiang SX, Kaboli PJ, Shen J, Zhao YS, et al. Engineered TCR-T cell immunotherapy in anticancer precision medicine: pros and cons. Front Immunol. 2021;12:658753. 10.3389/fimmu.2021.658753 PMC8042275 33859650

